# The involvement of Croatian psychiatrists in helping the displaced persons from Ukraine

**DOI:** 10.1192/j.eurpsy.2023.176

**Published:** 2023-07-19

**Authors:** M. Rojnic Kuzman

**Affiliations:** ^1^Zagreb University Hospital Centre; ^2^Zagreb School of Medicine, Zagreb, Croatia

## Abstract

**Abstract:**

After two years of pandemic with COVID-19 Europe is facing a war, which has already caused numerous death and injuries, mass displacement, and aggravated the economic and energy crisis and has left most countries completely unprepared and created a humanitarian crisis. The COVID-19 pandemics crisis pointed out the unpreparedness of the health (including mental health) sectors for the emergency situations. However, we also learnt some of the practices that proved effective – including the fast creation of collaborative networks on a larger scale that also allowed fast spread of good practices and practical organisation of help. The European Psychiatric Association as well as individual national psychiatric association have started an informal network of solidarity for Ukraine on February 28^th^, 2022 to respond to the needs of people in Ukraine as verbalized by the Ukrainian mental health professionals, but also to the need of surrounding countries where people from Ukraine fled to. Through this network several actions, including financial support, medical donations and education. The Croatian Psychiatric Association took the lead in the organisation of education for first line helpers and volunteers from Ukraine and countries surrounding Ukraine where displaced persons fled to, in collaboration with many partners.

**Disclosure of Interest:**

None Declared

